# Computed tomography derived segment involvement score and coronary artery calcium score when used in clinical routine—*data from a Swedish Registry Cohort*

**DOI:** 10.1093/ehjci/jeag090

**Published:** 2026-04-13

**Authors:** Henrik Löfmark, Ellen Ostenfeld, Tomasz Baron, Erika Fagman, Kari Feldt, Hanna Markstad, Josephine Muhrbeck, Mårten Sandstedt, Kambiz Shahgaldi, Eva Zelleroth, David Erlinge, Tomas Jernberg

**Affiliations:** Department of Clinical Sciences, Danderyd Hospital, Karolinska Institutet, Stockholm 182 88, Sweden; Department of Medical Imaging and Physiology, Cardiac Imaging, Skåne University Hospital, Lund University, Lund, Sweden; Department of Medical Sciences, Cardiology, and Uppsala Clinical Research Center, Uppsala University, Uppsala, Sweden; Department of Radiology, Institute of Clinical Sciences, Sahlgrenska Academy, University of Gothenburg, Gothenburg, Sweden; Department of Radiology, Region Västra Götaland, Sahlgrenska University Hospital, Gothenburg, Sweden; Department of Medicine, Solna, Karolinska Institutet, Stockholm, Sweden; Department of Medical Imaging and Physiology, Cardiac Imaging, Skåne University Hospital, Lund University, Lund, Sweden; Department of Clinical Sciences, Lund University, Malmö, Sweden; Department of Clinical Sciences, Danderyd Hospital, Karolinska Institutet, Stockholm 182 88, Sweden; Department of Radiology in Linköping and Department of Health, Medicine and Caring Sciences, Linköping University, Linköping, Sweden; Department of Clinical Sciences, Danderyd Hospital, Karolinska Institutet, Stockholm 182 88, Sweden; Department of Medicine, Malarsjukhuset, Eskilstuna, Sweden; Department of Clinical Sciences, Cardiology, Lund University, Lund, Sweden; Department of Clinical Sciences, Danderyd Hospital, Karolinska Institutet, Stockholm 182 88, Sweden

**Keywords:** calcium score, segmentation involvement score, coronary computed tomography angiography, coronary artery disease

## Abstract

**Aims:**

This study aimed to evaluate the prognostic value of segment involvement score (SIS) from coronary computed tomography angiography (CCTA) and compare it with coronary artery calcium score (CACS) in clinical practice.

**Methods and results:**

Patients undergoing CCTA for suspected coronary artery disease between 2006 and 2022 at 27 centres were included. SIS was defined by the number of segments with plaque. CACS was calculated using the Agatston method. Patients were followed for all-cause death and/or myocardial infarction (MI). A total of 23,034 patients were followed for a median of 2.5 years. SIS = 0 was found in 61.4% of patients, SIS = 1 in 12.6%, SIS = 2 in 8.2%, SIS = 3 in 5.7%, and SIS ≥ 4 in 12.2%. Compared with SIS = 0, SIS ≥ 4 was associated with higher adjusted risk of death (HR [95% CI]: 1.39 [1.17–1.66]), MI (3.53 [2.72–4.59]), and death or MI (1.88 [1.62–2.18]). Obstructive stenosis (≥50%) was also independently associated with all outcomes but showed lower discrimination than SIS in receiver operating characteristic curve analyses. SIS and CACS had similar ability to predict death or MI (Area under the curve: 0.70 [0.67–0.74] vs. 0.68 [0.65–0.72], *P* = 0.08) and MI alone (0.72 [0.67–0.77] vs. 0.72 [0.67–0.78], *P* = 0.69). CACS performed better than SIS in predicting death (0.70 [0.66–0.74] vs. 0.67 [0.63–0.70], *P* = 0.008).

**Conclusion:**

Both the extent of coronary atherosclerosis, measured by SIS, and the presence of obstructive disease are important predictors of outcomes. However, they do not provide additional prognostic value over CACS when used in routine clinical practice.

## Graphical Abstract

**Graphical Abstract:**
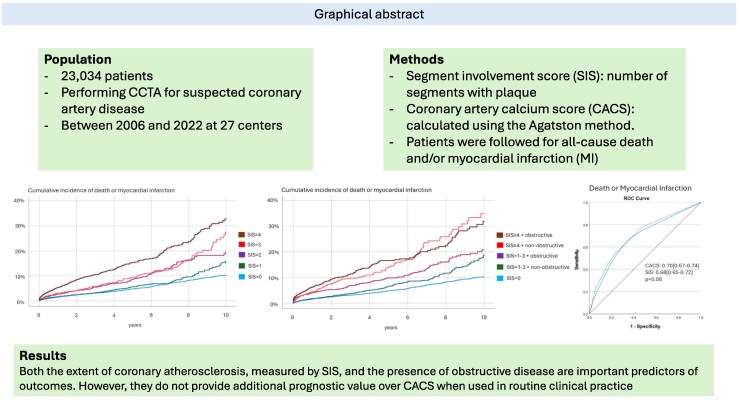

**See the editorial comment for this article ‘Old but gold: CACS vs. SIS in cardiac CT’, by E. Zsarnoczay and P. Maurovich-Horvat, https://doi.org/10.1093/ehjci/jeag108.**

## Introduction

Coronary artery disease remains the leading cause of death worldwide.^1–3^ After several decades of decreasing incidence, the decline in myocardial infarctions (MI) may have plateaued.^1–3^ Additionally, the prognosis following MI is no longer improving at the same rate, which has been observed particularly among younger individuals.^1–3^

Still, it is difficult to predict which individuals will suffer from acute MI. Risk scores, such as the Framingham risk score, Pooled Cohort Equation and the Systemic Coronary Risk Estimation 2 (SCORE2) are used in clinical practice to predict the 10-year risk of cardiovascular disease, defined as fatal or non-fatal MI or stroke.^4–6^ However, these scores lack precision, and the risk factors associated with MI and stroke differ.

Measurement of coronary artery calcium score (CACS) can be performed from a computed tomography (CT) examination and is a semi-automatic, quantitative method with high reproducibility. CACS improves risk stratification, when used as a complement to a score or used by its own to predict risk for future coronary events.^7–9^ A disadvantages of CACS is that it does not account for non-calcified atherosclerosis and obstructive coronary artery disease. In recent years, non-invasive evaluation with coronary computed tomography angiography (CCTA) has advanced rapidly and is today a routine method for visualizing coronary atherosclerosis.^10^ The segment involvement score (SIS), derived from visual assessment of CCTA, reflects the number of coronary segments with any type of atherosclerotic plaque and is a measure of overall disease burden. SIS has been shown to be an independent predictor of coronary events.^11^ However, SIS is a semi-quantitative measure of atherosclerosis, and interpretation of CCTA in clinical practice is usually performed manually by several different readers, which may introduce substantial inter-observer variability.

To date, few large studies have compared SIS and CACS in the context of routine clinical practice.^12–14^ The aim of this study was to examine the prognostic value of SIS, and to compare it with that of CACS, when used in everyday clinical settings without access to core laboratory reading, in a large patient cohort.

## Methods

Hospitals in Sweden performing CCTA register the examinations in the Swedish Web-system for Enhancement and Development of Evidence-based care in Heart disease Evaluated According to Recommended Therapies (SWEDEHEART) registry. In this study, patients undergoing CCTA between 2006 and 2022 for suspected coronary artery disease as the cause of their symptoms at 27 centres nationwide were included. We excluded patients with a history of revascularization—either percutaneous coronary intervention or coronary artery bypass graft surgery—or those lacking survival data due to residency outside of Sweden. For patients who underwent more than one CCTA, only the first examination was included in the analyses. All patients had been informed about their participation in the registry and their right to opt out; however, informed consent is not required for inclusion in quality registries. The study was approved by the Swedish ethical review authority (D-nr: 2024-02514-02). The individual data underlying this article cannot be shared publicly due to legal reasons.

Demographic information and baseline health status data were collected from the SWEDEHEART registry. Data entry into the registry was performed prospectively according to local standards, either through patient questionnaires or extraction from medical records at the time of scanning. Diabetes was considered present if the patient reported the use of anti-diabetic medications, including insulin. Hypertension was defined as the use of antihypertensive medication. Hyperlipidaemia was defined as the use of lipid lowering therapy. Serum or plasma creatinine levels were measured within 3 weeks prior to the scan. Body mass index (BMI) was calculated from self-reported height and weight. At each site, the interpreting physician was responsible for registering patient data and CCTA findings into the SWEDEHEART registry.

CCTA was performed using ≥64-slice multi-detector CT scanners with ECG-gated coronary angiography protocols, according to local discretion. CCTA exams with high heart rates and/or suboptimal image quality were processed using either multiphase reconstruction or multi-segment reconstruction, in accordance with each hospital’s protocol and the prevailing guidelines at that time of scanning.

For each coronary segment, findings were categorized as: no coronary artery disease (no plaque and no stenosis), non-obstructive stenosis (<50% luminal narrowing), or obstructive stenosis (≥50% luminal narrowing). Segments were marked as non-assessable either due to calcium blooming or technical issues.

Segments were defined according to the Society of Cardiovascular Computed Tomography and used in calculating the SIS score.^15^ Any coronary segments containing plaque, regardless of composition or size, was considered to have atherosclerotic disease. Segments deemed non-assessable due to calcium blooming were also categorized as having atherosclerotic disease. The total SIS score was calculated per patient and reported as the exact score if <4 or as ≥4 for higher burden.

For CACS, we used the scoring algorithm proposed by Agatston *et al*.^16^ Non-contrast CT images with a 3 mm slice thickness were acquired across the entire cardiac field. At least 3 contiguous pixels exceeding 130 Hounsfield units HU defined a calcific lesion and assigned a numerical score to the corresponding area. The total CACS was the sum of all scores constituted the participant’s final score and was reported numerically in the registry. In this study, CACS was categorized as follows: 0 (none), 1–9 (minimal), 10–99 (mild), 100–299 (moderate) and ≥300 (severe).

Patients were followed for up to 10 years. The primary endpoint was defined as all-cause mortality or MI, with only the first MI after CCTA considered in cases of multiple events. Information on mortality was obtained from the Swedish population registry which includes the vital status of all Swedish residents. Data on MI or hospital admissions for MI were collected from SWEDEHEART, covering all Swedish hospitals with acute cardiac care.

### Statistics

The continuous variables are presented as mean ± SD or median [interquartile range], comparison between groups was performed using the unpaired *t*-test, the Mann–Whitney test or the Kruskal–Wallis test. The categorical variables are presented as frequencies with percentages based on non-missing values and a Chi-square test or Fisher’s exact test was used for comparison between groups. Cumulative incidence of death or readmission because of MI, death alone and MI readmission alone censoring for death was illustrated with the Kaplan–Meier method.

Cox proportional hazards models were used to assess the relationship of assigned SIS, CACS and outcomes without and with adjustment for background variables. Adjustments were made for age, sex, smoking, diabetes mellitus, hypertension, hyperlipidaemia, BMI, estimated glomerular filtration rate and previous MI. To handle missing values, multiple imputations was performed with 5 complete data sets. The assumption of proportional hazards was tested using visual inspection and scaled Schoenfeld residuals. To compare the prognostic value of SIS and CACS, receiver-operating characteristic (ROC) curves were generated and area under curves (AUC) were calculated and compared. SPSS version 28 (IBM, Armonk, NY, USA) and R 4.4.1 and R Studio, version 2024.04.1 were used for statistical analyses.

## Results

### Baseline data

A total of 36,168 CCTA examinations were registered between 18 January 2006 and 16 February 2022, with a gradual increase in registered examinations (see Supplementary data online, *Figure S1*). After excluding patients with more than one examination (*n* = 1519), previously revascularized (*n* = 2918), non-residences with no follow-up data (*n* = 642), patients undergoing CCTA for other reasons than suspected coronary artery disease (*n* = 7595) and those not receiving contrast (*n* = 460), the remaining 23,034 patients were included in the analysis (see Supplementary data online, *Figure S2*). The median age was 58 years, 50.5% were male, 11.8% were current smokers, 9.3% had diabetes mellitus, and 2.5% had a history of previous MI (*Table 1*). Missing data are shown in Supplementary data online, *Table S1*.

**Table 1:** Patient characteristic in all patients in the analyses and in relation to segment involvement score

	SIS group					
Characteristics	All *n* = 23,034	SIS = 0 *n* = 14,145	SIS = 1 *n* = 2894	SIS = 2 *n* = 1880	SIS = 3 *n* = 1302	SIS ≥ 4 *n* = 2813
Age, years	58 [49–67]	55 [46–64]	60 [52–68]	63 [55–70]	64 [57–72]	66 [59–72]
Male sex	11,625 (50.5%)	6406 (45.3%)	1507 (52.1%)	1071 (57.0%)	761 (58.4%)	1880 (66.8%)
Smoking, current	2285 (11.8%)	1310 (10.9%)	291 (12.0%)	204 (12.7%)	149 (13.3%)	331 (14.7%)
Diabetes	2039 (9.3%)	926 (6.9%)	248 (9.0%)	221 (12.3%)	199 (16.0%)	445 (16.7%)
Hypertension	9722 (48.0%)	5165 (41.4%)	1309 (51.2%)	945 (56.9%)	719 (62.1%)	1584 (65.8%)
Hyperlipidaemia	5725 (29.0%)	2901 (23.8%)	743 (29.8%)	570 (35.4%)	471 (41.7%)	1040 (45.7%)
BMI, kg/m^2^	26.3 [23.7–29.7]	26.2 [23.5–29.4]	26.3 [23.7–29.7]	26.5 [23.9–30.0]	26.6 [24.2–29.9]	26.9 [24.2–30.1]
eGFR, mL/min/1.73 m^2^	93.5 [74.9–116.8]	96.9 [77.6–120.7]	91.5 [73.3–112.5]	90.1 [71.5–111.8]	87.5 [70.7–111.2]	86.0 [69.1–107.3]
Previous MI	529 (2.5%)	281 (2.1%)	50 (1.9%)	43 (2.5%)	37 (3.0%)	118 (4.6%)
Obstructive stenosis (≥50%)	2959 (12.8%)	0 (0%)	560 (19.4%)	489 (19.4%)	487 (37.4%)	1423 (50.6)
Calcium score 0	4145 (50.4%)	3802 (89.5%)	244 (20.5%)	66 (8.4%)	18 (3.3%)	15 (1.0%)
Calcium score 1–9	750 (9.1%)	177 (4.2%)	362 (30.5%)	137 (17.5%)	51 (9.3%)	23 (1.6%)
Calcium score 10–99	1580 (19.2%)	147 (3.5%)	483 (40.7%)	409 (52.2%)	253 (46.1%)	288 (19.8%)
Calcium score 100–299	878 (10.7%)	56 (1.3%)	72 (6.1%)	138 (17.6%)	152 (27.7%)	460 (31.6%)
Calcium score ≥300	872 (10.6%)	66 (1.6%)	27 (2.3%)	34 (4.3%)	75 (13.7%)	670 (46.0%)

Data expressed as median [IQR] or numbers and proportion (%).

BMI, Body mass index; eGFR, Estimated glomerular filtration rate; MI, Myocardial infarction; SIS, Segment involvement score.

The distribution of SIS scores is shown in Supplementary data online, *Figure S3A*. No coronary atherosclerosis (SIS = 0) was observed in 61.4% of patients, while 12.6% had SIS = 1, 8.2% SIS = 2, 5.7% SIS = 3, and 12.2% SIS ≥ 4. Obstructive disease (≥50% stenosis) was present in 2959 (12.8%) patients. Among the 19 hospitals registering more than 100 examinations, there was a large variation in SIS distribution, with the proportion of patients with SIS = 0 ranging from 42.0% to 98.6%, and those with SIS ≥4 ranging from 0.7% to 25.6% (see Supplementary data online, *Figure S4*). With increasing SIS, patients tended to be older, more often male and current smokers, and had higher BMI, lower eGFR, and higher prevalence of diabetes mellitus, hypertension, hyperlipidaemia, and previous MI (*Table 1*).

### Associations between CCTA findings and outcome

During a median follow-up of 2.50 [IQR 1.05–5.13] years, 1231 patients (5.3%) experienced the primary outcome of death or MI; 900 patients (3.9%) died, and 394 (1.7%) experienced MI. The incidence rate per 1000 person-years was 15.8 [95%CI 14.9 −16.7] for the composite outcome of death or MI, 11.4 [95%CI 10.6 −12.1] for death and 5.1 [95%CI 4.6 −5.6] for MI.

**Figure 1:**
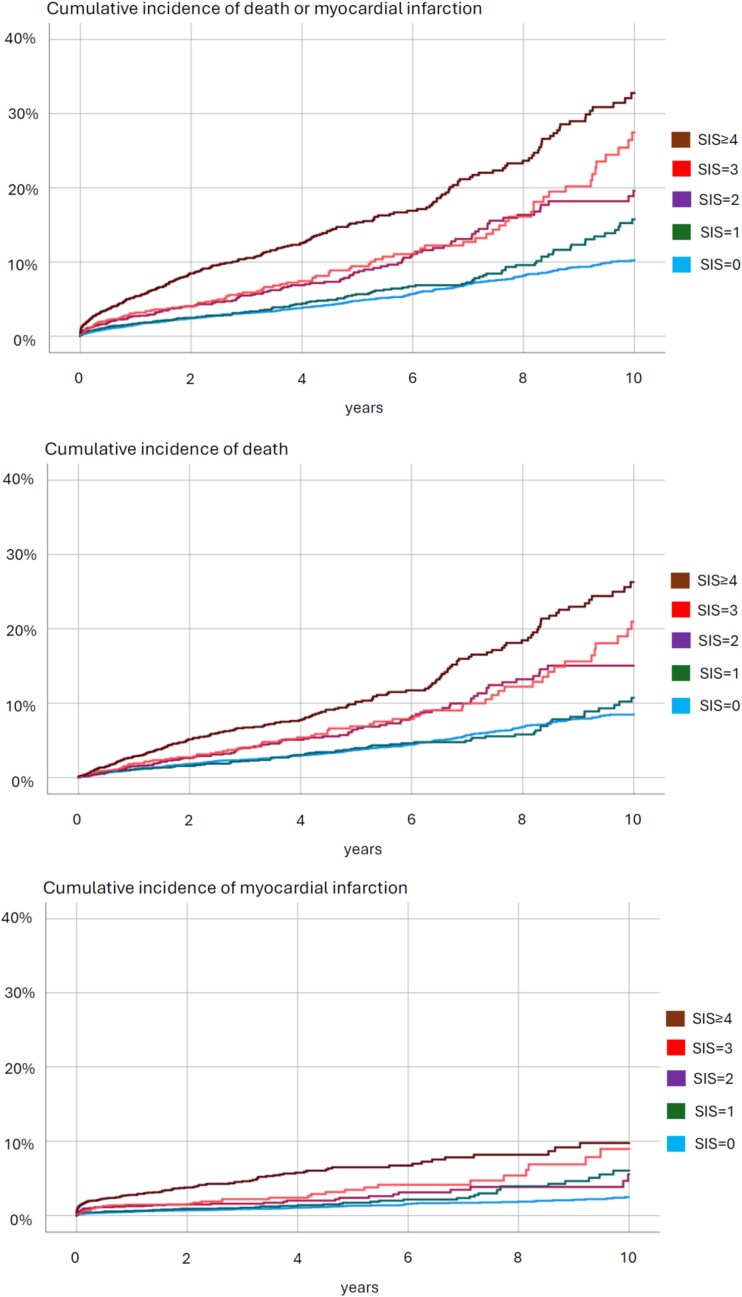
Cumulative incidence of death or myocardial infarction (upper panel), death alone (middle panel), and myocardial infarction alone (lower panel), in relation to SIS.

**Figure 2:**
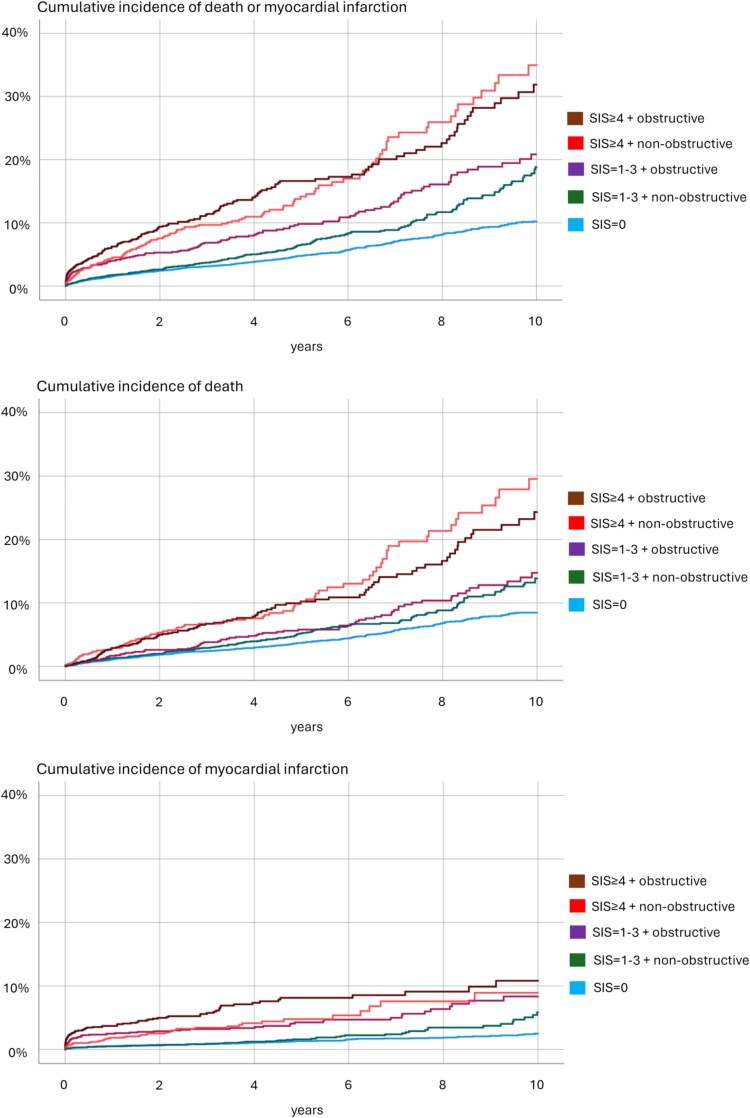
Cumulative incidence of death or myocardial infarction (upper panel), death alone (middle panel), and myocardial infarction alone (lower panel), in relation to SIS and obstructive coronary artery disease stenosis ≥50%.

The cumulative incidence of death or MI, death alone and MI alone increased with higher SIS (*Figure 1*) and with presence of obstructive stenosis (see Supplementary data online, *Figure S5*). These associations remained statistically significant after adjustment for baseline characteristics, and also when presence of obstructive stenosis was included in the model (*Table 2*). When categorizing individuals into those with: (i) no coronary atherosclerosis, (ⅱ) SIS = 1–3 and no obstructive stenosis (<50%), (ⅲ) SIS = 1–3 and obstructive stenosis (≥50%), (ⅳ) SIS ≥4 and no obstructive stenosis, and (ⅴ) SIS ≥4 and obstructive stenosis, SIS was associated with all three outcomes regardless of the presence of obstruction (*Figure 2*, Supplementary data online, *Table S2*). Obstructive stenosis (≥50%) was independently associated with all three outcomes when adjusting for baseline characteristics (see Supplementary data online, *Table S3*) and with death or MI and MI alone when SIS was included in the model (*Table 2*). When SIS and number of segments showing ≥50% stenosis were compared, SIS showed significantly higher discriminatory ability for death or MI (AUC: 0.616 vs. 0.572, *P* < 0.001), death alone (AUC: 0.596 vs. 0.549, *P* < 0.001), and MI alone (AUC: 0.656 vs. 0.623, *P* = 0.006). Combining SIS and the number of segments with ≥50% stenosis did not improve predictive performance (see Supplementary data online, *Table S4*).

**Table 2:** Segment involvement score, stenosis ≥50% and outcome

	Unadjusted		Adjusted^a^	
	HR	95% CI	HR	95% CI
**Death or myocardial infarction**				
SIS = 0	1	Ref	1	Ref
SIS = 1	1.23	1.02–1.49	0.97	0.80–1.17
SIS = 2	1.91	1.58–2.31	1.25	1.03–1.52
SIS = 3	2.20	1.78–2.71	1.31	1.05–1.62
SIS ≥ 4	3.54	3.08–4.07	1.88	1.62–2.18
**Death**				
SIS = 0	1	Ref	1	Ref
SIS = 1	1.04	0.83–1.30	0.79	0.63–0.99
SIS = 2	1.75	1.40–2.18	1.07	0.86–1.34
SIS = 3	1.98	1.55–2.54	1.07	0.83–1.38
SIS ≥ 4	2.96	2.52–3.50	1.39	1.17–1.66
**Myocardial infarction**				
SIS = 0	1	Ref	1	Ref
SIS = 1	1.62	1.17–2.24	1.40	1.01–1.95
SIS = 2	2.10	1.47–2.99	1.66	1.15–2.37
SIS = 3	2.85	1.98–4.10	2.12	1.45–3.08
SIS ≥ 4	5.12	4.03–6.50	3.53	2.72–4.59
**Death or myocardial infarction**				
SIS = 0	1	Ref	1	Ref
SIS = 1	1.17	0.96–1.41	0.93	0.77–1.13
SIS = 2	1.76	1.45–2.15	1.18	0.97–1.44
SIS = 3	1.99	1.59–2.48	1.21	0.97–1.52
SIS ≥ 4	3.09	2.62–3.65	1.70	1.43–2.02
Stenosis ≥50%	1.27	1.09–1.48	1.21	1.03–1.41
**Death**				
SIS = 0	1	Ref	1	Ref
SIS = 1	1.04	0.83–1.31	0.81	0.64–1.02
SIS = 2	1.76	1.40–2.22	1.10	0.88–1.39
SIS = 3	2.00	1.55–2.59	1.11	0.86–1.44
SIS ≥ 4	3.01	2.48–3.65	1.47	1.20–1.79
Stenosis ≥50%	0.97	0.80–1.18	0.91	0.75–1.10
**Myocardial infarction**				
SIS = 0	1	Ref	1	Ref
SIS = 1	1.32	0.94–1.85	1.15	0.82–1.63
SIS = 2	1.60	1.10–2.32	1.27	0.97–1.86
SIS = 3	2.02	1.36–2.99	1.53	1.02–2.29
SIS ≥ 4	3.27	2.42–4.41	2.33	1.70–3.19
Stenosis ≥50%	2.07	1.60–2.69	2.04	1.57–2.65

SIS, Segment involvement score.

^a^Adjusted for age, sex, smoking, diabetes mellitus, hypertension, hyperlipidaemia, BMI, eGFR and previous myocardial infarction.

### Comparison between SIS and CACS

Among the 8225 patients who underwent both CCTA and CACS measurement, baseline characteristics were similar to the 23,034 patients undergoing CCTA (see Supplementary data online, *Table S5*). The distributions of SIS and CACS scores for this subgroup are shown in Supplementary data online, *Figure S3B* and *C*. The cumulative incidence of all outcomes increased with increasing SIS and CACS (see Supplementary data online, *Figure S6*). When patients were grouped using SIS categories (0–1, 2, 3, ≥4) and similarly sized CACS categories, the outcome curves appeared similar with similar separation of the groups both in the univariable and the multivariable analyses (see Supplementary data online, *Figure S7* and *Table S6*).

**Figure 3:**
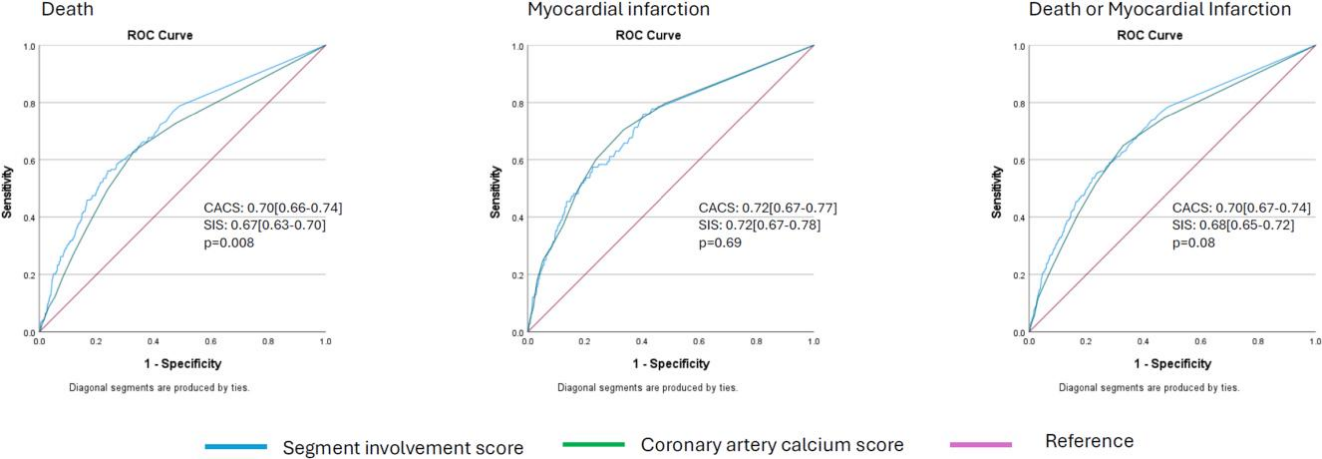
ROC curves showing the ability of SIS and CACS to discriminate between patients with subsequent death, myocardial infarction, and death or myocardial infarction.

In a receiver operating characteristic analysis, CACS and SIS had similar ability to discriminate the risk of death or MI and MI alone, while CACS showed better discrimination for death alone (*Figure 3*). Combining SIS with CACS did not improve discriminatory ability compared with CACS alone (see Supplementary data online, *Table S7*).

## Discussion

We could demonstrate that the burden of coronary atherosclerosis, measured by SIS, is a strong predictor of death or MI, as well as death and MI alone, even when SIS was applied in routine clinical practice across diverse laboratories and readers nationwide, without core-lab validation. A SIS ≥4 was associated with a fivefold increased risk of MI and a threefold increased risk of death compared with individuals without coronary atherosclerosis. SIS was a stronger predictor of outcomes than the presence of obstructive coronary artery disease, defined as ≥50% luminal stenosis. However, when directly compared with CACS, SIS and CACS showed similar prognostic value for death or MI and MI alone, while CACS showed a modestly better ability to predict death. Combining SIS with CACS did not improve discriminatory ability.

This study includes a large proportion of the CCTA examinations performed in Sweden from the start of registrations in 2006 to 2022, demonstrating a continuous increase in their clinical utilization, from 500 exams in 2008 to about 7000 in 2021. More than 60% showed no signs of coronary atherosclerosis, 12% had a SIS of 4 or more, and 13% had obstructive disease. Except for a higher proportion of individuals with obstructive disease (13% vs. 5%), CCTA findings were very similar to those in the Swedish CArdioPulmonary BioImage Study (SCAPIS), which involved a general population of similar median age.^17^ This indicates that CCTA has primarily been used in low-risk patients to exclude coronary artery disease. However, substantial inter-centre variations existed: the proportion of patients without atherosclerosis ranged from 42% to 99% and SIS ≥4 was present in 1% to 26% of the examined patients, highlighting differences in case selection, reading practices, or both.

Patients were followed up for up to 10 years with increasing risk of death or MI with increasing SIS. The cumulative incidence of death or MI was 10% in individuals with no atherosclerosis and 32% in those with SIS ≥4. After adjusting for risk factors and comorbidities, a SIS ≥4 remained associated with a 50% increased risk of all-cause death, a doubled risk of death or MI and a threefold increase in MI alone. The association between less severe atherosclerosis (SIS 1–3) and death was, however, attenuated and not statistically significant after adjustment. Severe atherosclerosis (SIS ≥4) was more strongly associated with death than presence of obstruction (stenosis ≥50%), whereas both the extent and presence of obstruction provided prognostic information regarding the risk of MI and death or MI. These findings may not be generalized to other populations or settings. Our results are, however, in line with several other studies demonstrating that both the extent and the presence obstruction are associated with subsequent outcome.^11^ The results also support results from the Copenhagen General Population Study, which followed 9533 asymptomatic individuals for a median of 3.5 years after a core-lab read CCTA.^18^ Extensive disease was associated with an increased risk of death or MI, regardless of whether the disease was obstructive. The risk of MI was linked to both the extent of the disease and the presence of obstruction, with the strongest association observed for obstruction, which is supported by our study. However, when SIS and number of segments with stenosis ≥50% were compared in a ROC analysis in our study, SIS had a significantly larger AUC for all three studied endpoints, which suggests a better predictive ability for SIS in the clinical setting. In contrast, the SCOT-HEART study and a study by van den Hoogen *et al*., both using core-lab readings, demonstrated no differences in AUCs for the discrimination of MI or major adverse cardiovascular events when the prognostic value of SIS was compared with segment stenosis score and other scores taking obstructive stenosis into account.^19,20^

When SIS and CACS were compared, they had similar ability to discriminate between death or MI and MI alone, whereas CACS was superior in predicting all-cause mortality. Combining SIS with CACS did not add any discriminatory ability compared with CACS alone. These findings are also in line with some previous findings. Like in the CONFIRM study including clinical cases from several different centres and following patients regarding all-cause mortality, CCTA findings did not have additional prognostic value compared with information obtained from risk factors and CACS.^21^ Furthermore, in the multicentre study, CORE320, CACS had an additive value to predict cardiac death, MI or stroke compared with risk factors alone, but addition of more complex measurements of coronary atherosclerotic burden did not improve prognostication.^22^

There are clinical implications of this study that need to be considered. In patients with suspected symptomatic ischaemic heart disease, CCTA is the preferred modality over CACS, as it provides information on the presence of obstructive disease. Our study confirmed that CCTA could substantially improve risk stratification when added to risk factors and comorbidities in the clinical routine. However, during the early adoption of CCTA in Sweden, substantial variability in reported findings was evident, likely reflecting more than just differences in patient populations, and CCTA data did not provide additional prognostic value beyond CACS. This highlights the need for quality improvement efforts, including measures to reduce inter-observer variability and to promote the use of tools for more reproducible quantitative plaque analysis compared with manual readings. Recently, Nurmohamed *et al*.^23^ demonstrated that artificial intelligence-guided quantitative CCTA analysis of plaque burden can enhance the prediction of cardiovascular events beyond traditional risk factors and CACS.

Our study has several strengths and limitations. Its large sample size and long-term follow-up, with a substantial number of events, enables comparisons of the prognostic value of multiple factors and an assessment of the additive value of one factor over another. Another key strength is the use of real-world registry data, capturing the development of the method from its inception across an entire country. This includes readings from multiple centres as they built expertise in performing CCTA, ensuring a high degree of generalizability. This contrasts with other studies where the selection of centres and patients may be less clear. The present study has limitations. These were clinical examinations and may therefore have guided downstream management of patients, particularly those with severe atherosclerosis and obstructive disease, which could have influenced the association between findings and subsequent outcome. This is a common limitation for this type of study. CACS measurement was from start not part of the clinical routine at many centres, limiting comparisons between SIS and CACS to a subgroup of patients. Nonetheless, this comparison included over 8000 examinations, exceeding the size of many previous studies. We cannot exclude that examinations were marked as ‘normal’ due paradigm shifts in the guidelines during the inclusion period. Registration has over the years changed focus from emphasizing obstructive stenosis or not to more focus on registering any segment with a plaque even when non-obstructive. Furthermore, although likely uncommon, we cannot exclude some degree of registration ‘fatigue’ in the SWEDEHEART registry. Underregistration of non-obstructive plaque would lead to an underestimation of the predictive value of CCTA. Missing values were present for some variables in the adjusted analyses, such as smoking (up to 20%), potentially introducing selection bias. However, this risk was mitigated by using multiple imputation. Other types of plaque characterization were not included due to limited reporting of plaque composition and high likelihood of substantial inter-observer variability across centres. Additionally, we used all-cause death as part of the outcome rather than coronary heart death, likely leading to an underestimation of the prognostic value of CCTA.

In conclusion, both the extent of coronary atherosclerosis, measured as SIS, and the presence of obstructive stenosis are important predictors of adverse outcomes. However, they do not provide additional prognostic value compared with CACS in long-term follow-up when used in routine clinical practice, as observed in Sweden during the implementation of this new diagnostic method.

## Supplementary Material

jeag090_Supplementary_Data

## Data Availability

Because of data protection regulations, the authors do not have the authorization to provide unrestricted data access. Requests for data and additional documents related to the study should be made to the corresponding author.
