# Supplementary material for: Computed tomography derived segment involvement score and coronary artery calcium score when used in clinical routine—data from a Swedish Registry Cohort
Source: Eur Heart J Cardiovasc Imaging. 2026 Apr 13;27(7):1345–54. doi: 10.1093/ehjci/jeag090 (PMC13314024; doi:10.1093/ehjci/jeag090)
Supplement: jeag090_Supplementary_Data [file jeag090_supplementary_data.docx]

**Supplementary Material (Löfmark et al)**

**Figure S1,** Numbers of registered coronary computed tomography angiographies per year

**Figure S2,** Flowchart, iluustrating patient selection

CCTA examinations during Jan 2006 to Feb 2022 (n=36,168)

More than one examination (n=1,519) )555519519519)

Previous revascularization (n=2,918)

No survival data (n=642)

Other indications (n=7,595)

No contrast (n=460)

No CACS reported (n=14,809)

*SIS Cohort* (n=23,034)

*SIS and CACS Cohort* (n=8,225)

*SIS Cohort:* SIS score assigned to all patients. *SIS and CACS Cohort:* Both SIS and CACS determined

**Figure S3,** Numbers of individuals assigned SIS and/or CACS

**Figure S4,** Distribution of SIS among different centers

**Figure S5,** Cumulative risk of death or MI (upper panel), dealth alone (middle panel) and MI alone (lower panel) in relation to presence of obstructive disease.


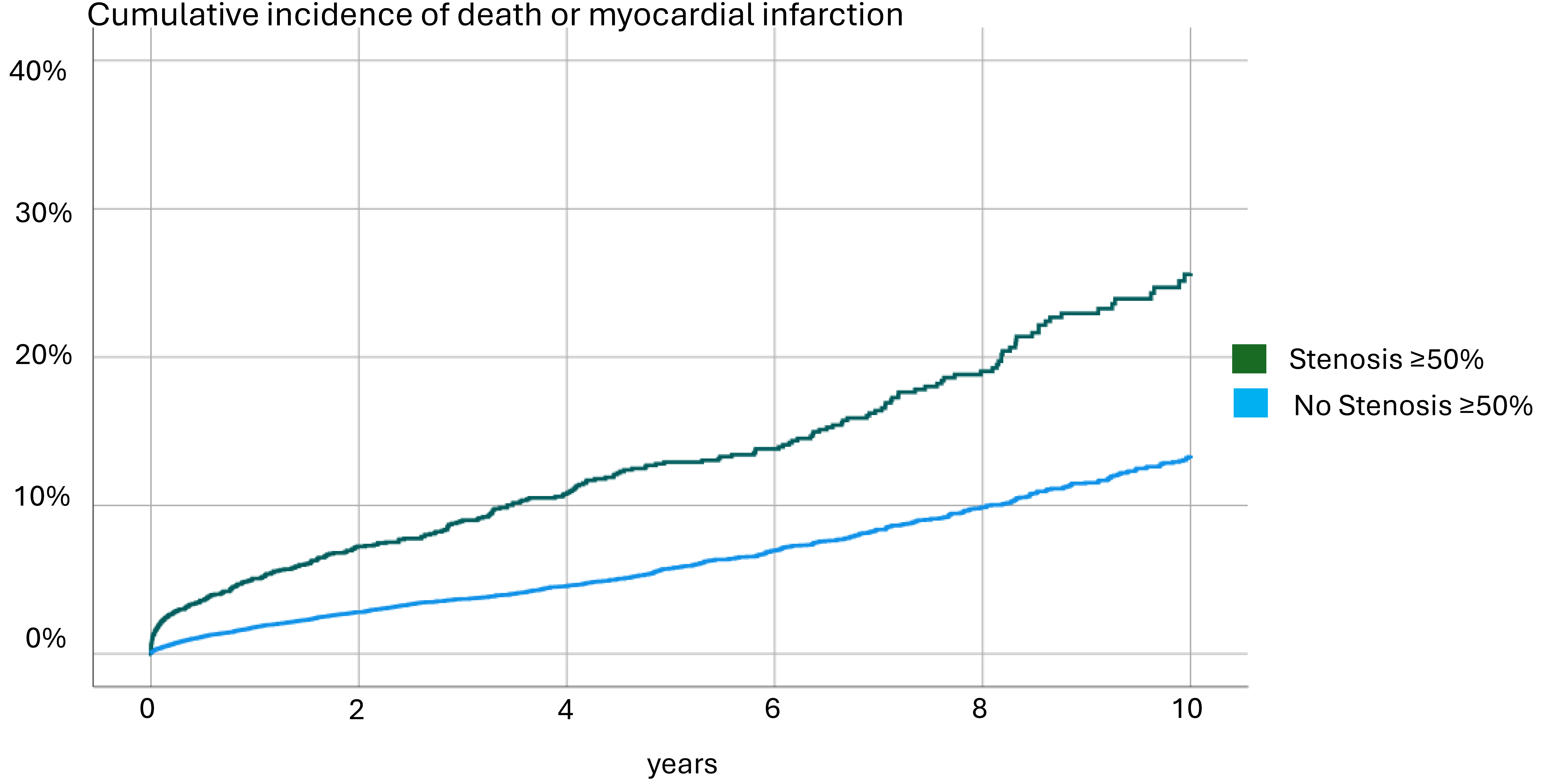


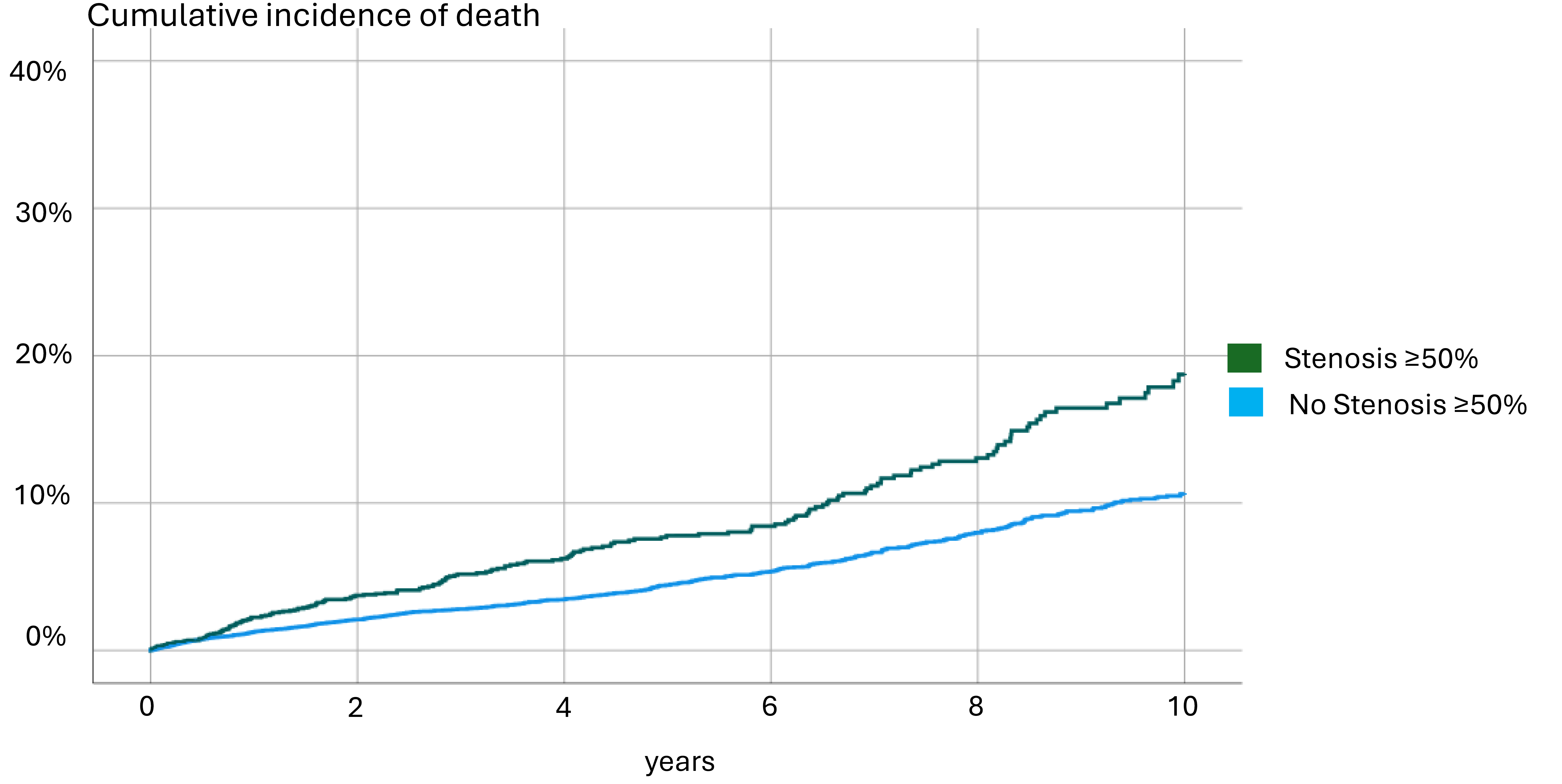


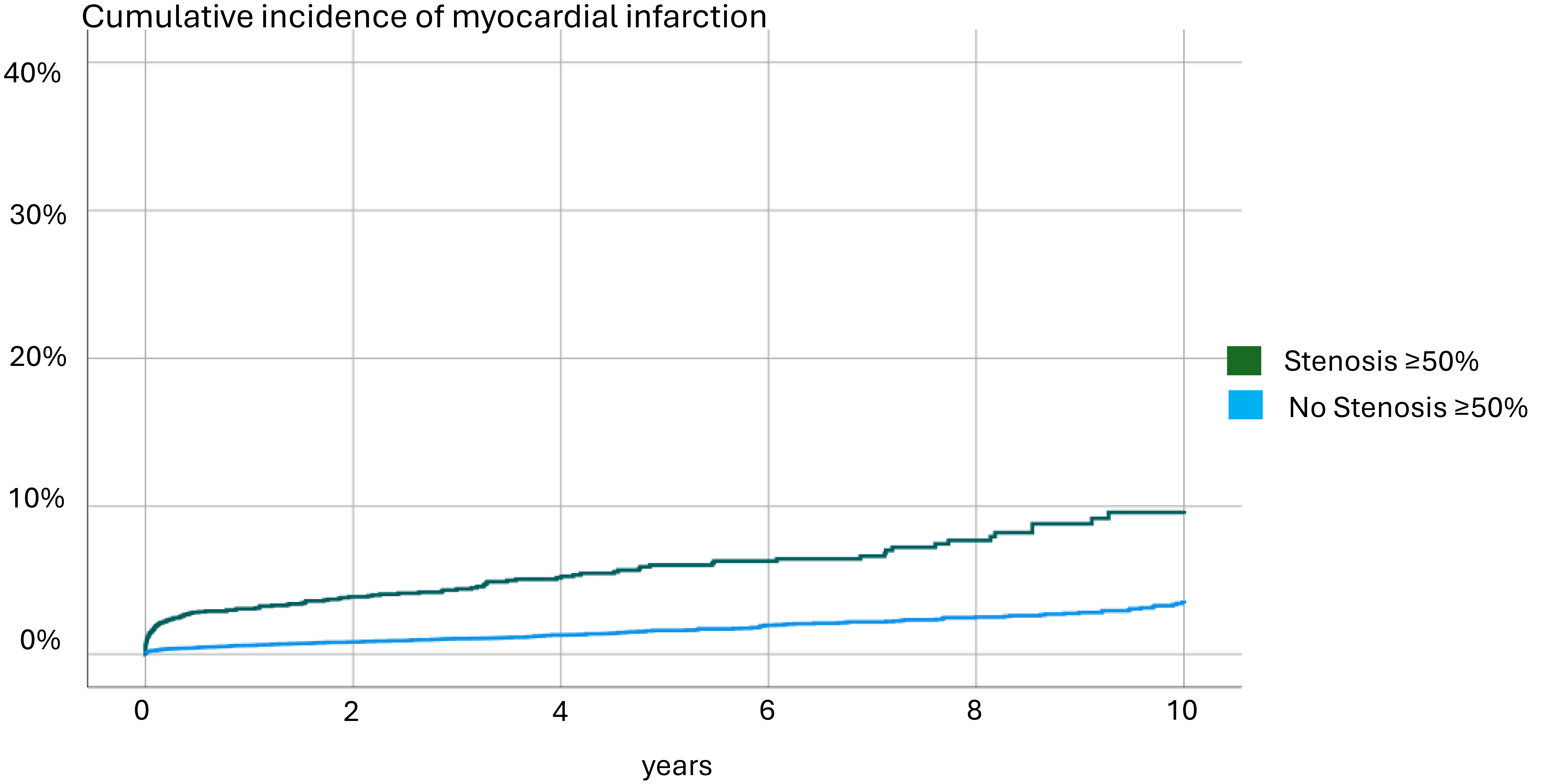


******Figure S6,** Cumulative risk of death or MI (upper panels), dealth alone (middle panels) and MI alone (lower panels) in relation to segment involvement score (SIS) and coronary artery calcium score (CACS).

**Figure S7,** Number of individuals grouped by coronary artery calcium score (CACS) in similar size as segment involvement score (SIS) categories (upper planel), cumulative risk of death or myocardial infarction in relation to SIS (middle panel) and CACS (lower panel).

**Table S1,** Patient characteristics – Missing values and unknown

|  | **SIS-group (n=23,034)** | | **SIS and CACS-group (n=8525)** | |
| --- | --- | --- | --- | --- |
| **Characteristics** | **Missing** | **Unknown** | **Missing** | **Unknown** |
| Age, median [25-75] | 0 | 0 | 0 | 0 |
| Male sex, n | 0 | 0 | 0 | 0 |
| Smoking, current, n | 55 | 3600 | 38 | 1681 |
| Diabetes, n | 42 | 1020 | 28 | 601 |
| Hypertension, n | 44 | 2734 | 24 | 1331 |
| Hyperlipidemia, n | 56 | 3259 | 36 | 1594 |
| BMI, median [25-75] | 4529 | 0 | 1959 | 0 |
| eGFR, ml/min/1.73 m^2^ | 6741 | 0 | 2743 | 0 |
| Previous MI, n | 32 | 1725 | 14 | 612 |
| Obstructive stenosis (≥50%) | 0 | 0 | 0 | 0 |
| CACS | 14509 | 14509 | 0 | 0 |

SIS: Segment involvement score, CACS: Coronary artery calcium score, BMI: Body mass index, eGFR: Estimated glomerular filtration rate, MI: Myocardial infarction.

**Table S2,** Association between segement involvement score and outcome in patients with non-obstructive and obstructive coronary artery disease

|  | **Unadjusted** | | **Adjusted*** | |
| --- | --- | --- | --- | --- |
|  | HR | 95%CI | HR | 95%CI |
|  |  |  |  |  |
| **Non-obstructive** |  |  |  |  |
|  |  |  |  |  |
| **Death or myocardial infarction** | | | | |
| SIS=1-3 | 1 | ref | 1 | ref |
| SIS≥4 | 2.42 | (1.96-2.99) | 2.07 | (1.60-2.68) |
|  |  |  |  |  |
| **Death** | | | | |
| SIS=1-3 | 1 | Ref | 1 | Ref |
| SIS≥4 | 2.33 | (1.83-2.97) | 1.88 | (1.39-2.53) |
|  |  |  |  |  |
| **Myocardial infarction** | | | | |
| SIS=1-3 | 1 | Ref | 1 | Ref |
| SIS≥4 | 2.88 | (1.96-4.24) | 2.93 | (1.81-4.75) |
|  |  |  |  |  |
| **Obstructive** |  |  |  |  |
|  |  |  |  |  |
| **Death or myocardial infarction** | | | | |
| SIS=1-3 | 1 | Ref | 1 | Ref |
| SIS≥4 | 1.65 | (1.32-2.06) | 1.40 | (1.09-1.82) |
|  |  |  |  |  |
| **Death** |  |  |  |  |
| SIS=1-3 | 1 | Ref | 1 | Ref |
| SIS≥4 | 1.36 | (1.31-2.32) | 1.36 | (0.98-1.89) |
|  |  |  |  |  |
| **Myocardial infarction** |  |  |  |  |
| SIS=1-3 | 1 | Ref | 1 | Ref |
| SIS≥4 | 1.43 | (1.18-2.28) | 1.43 | (0.98-2.09) |
|  |  |  |  |  |

SIS: Segment involvement score. *Adjusted for age, sex, smoking, diabetes mellitus, hypertension, hyperlipidemia, body mass index, estimated glomerular filtration rate and previous myocardial infarction. Complete case analysis.

**Table S3,** Association between obstructive coronary artery disease and outcome in all patients and in those with segment involvement score 1-3 and ≥4.

|  | **Unadjusted** | | **Adjusted** | |
| --- | --- | --- | --- | --- |
|  | HR | 95%CI | HR | 95%CI |
|  |  |  |  |  |
| **All** |  |  |  |  |
|  |  |  |  |  |
| **Death or myocardial infarction** | | | | |
| Non-obstructive | 1 | ref | 1 | ref |
| Obstructive | 1.99 | (1.66-2.39) | 1.48 | (1.21-1.82) |
|  |  |  |  |  |
| **Death** | | | | |
| Non-obstructive | 1 | ref | 1 | ref |
| Obstructive | 1.99 | (1.66-2.39) | 1.48 | (1.21-1.82) |
|  |  |  |  |  |
| **Myocardial infarction** | | | | |
| Non-obstructive | 1 | ref | 1 | ref |
| Obstructive | 1.99 | (1.66-2.39) | 1.48 | (1.21-1.82) |
|  |  |  |  |  |
| **SIS=1-3** |  |  |  |  |
|  |  |  |  |  |
| **Death or myocardial infarction** | | | | |
| Non-obstructive | 1 | ref | 1 | ref |
| Obstructive | 1.99 | (1.66-2.39) | 1.48 | (1.21-1.82) |
|  |  |  |  |  |
| **Death** | | | | |
| Non-obstructive | 1 | Ref | 1 | Ref |
| Obstructive | 1.48 | (1.18-1.86) | 1.03 | (0.80-1.34) |
|  |  |  |  |  |
| **Myocardial infarction** | | | | |
| Non-obstructive | 1 | Ref | 1 | Ref |
| Obstructive | 3.37 | (2.53-4.49) | 2.87 | (2.10-3.94) |
|  |  |  |  |  |
| **SIS≥4** |  |  |  |  |
|  |  |  |  |  |
| **Death or myocardial infarction** | | | | |
| Non-obstructive | 1 | Ref | 1 | Ref |
| Obstructive | 1.12 | (0.90-1.40) | 1.13 | (0.87-1.48) |
|  |  |  |  |  |
| **Death** |  |  |  |  |
| Non-obstructive | 1 | Ref | 1 | Ref |
| Obstructive | 0.88 | (0.67-1.15) | 0.87 | (0.63-1.20) |
|  |  |  |  |  |
| **Myocardial infarction** |  |  |  |  |
| Non-obstructive | 1 | Ref | 1 | Ref |
| Obstructive | 1.73 | (1.20-2.48 | 1.77 | (1.14-2.75) |
|  |  |  |  |  |

SIS: Segment involvement score. *Adjusted for age, sex, smoking, diabetes mellitus, hypertension, hyperlipidemia, body mass index, estimated glomerular filtration rate and previous myocardial infarction. Complete case analysis.

**Table S4,** Ability of segment involvement score (SIS), segment stenosis score (SSS) and combination of SIS and SSS (SIS x SSS) to discriminate between patients with subsequent composite of death or myocardial infarction, death alone and myocardial infarction alone.

|  | SIS | | SSS | | SIS x SSS | |
| --- | --- | --- | --- | --- | --- | --- |
|  | AUC | (95%CI) | AUC | (95%CI) | AUC | (95%CI) |
| Death or Myocardial infarction | 0.62 | (0.60-0.63) | 0.57 | (0.55-0.59) | 0.57 | (0.55-0.59) |
| Death | 0.60 | (0.58-0.62) | 0.55 | (0.53-0.57) | 0.55 | (0.53-0.57) |
| Myocardial infarction | 0.66 | (0.63-0.69) | 0.62 | (0.62-0.66) | 0.62 | (0.62-0.66) |

**Table S5a,** Patient characteristics in the SIS and CACS group

|  | **SIS group** | | | | | |
| --- | --- | --- | --- | --- | --- | --- |
| **Characteristics** | **Overall**, N = 8,225 | **0**, N = 4,248 | **1**, N = 1,188 | **2**, N = 784 | **3**, N = 549 | **≥4**, N = 1,456 |
| Age, median [25-75] | 58.0 [49.0-66.0] | 52.0 [44.0-61.0] | 59.0 [51.0-67.0] | 62.0 [54.0-68.0] | 63.0 [56.0-70.0] | 66.0 [59.0-72.0] |
| Male sex, n (%) | 4,074 (49.5%) | 1,780 (41.9%) | 598 (50.3%) | 440 (56.1%) | 299 (54.5%) | 957 (65.7%) |
| Smoking, current, n (%) | 850 (13.1%) | 401 (12.1%) | 119 (12.9%) | 87 (13.8%) | 59 (13.1%) | 184 (15.6%) |
| Diabetes, n (%) | 721 (9.5%) | 212 (5.5%) | 103 (9.4%) | 85 (11.6%) | 90 (17.5%) | 231 (16.9%) |
| Hypertension, n (%) | 3,152 (45.9%) | 1,234 (35.3%) | 488 (48.5%) | 357 (54.2%) | 277 (59.8%) | 796 (63.8%) |
| Hyperlipidemia, n (%) | 1,814 (27.5%) | 640 (18.9%) | 251 (26.3%) | 197 (31.4%) | 198 (44.2%) | 528 (44.6%) |
| BMI, median [25-75] | 26.4 [23.7-29.7] | 26.0 [23.4-29.4] | 26.4 [23.7-30.0] | 26.5 [23.9-29.9] | 26.8 [24.6-30.6] | 27.1 [24.3-30.4] |
| eGFR, mL/min/1.73 m^2^ | 96.0 [77.0-118.5] | 100.8 [81.3-124.9] | 92.8 [76.0-115.5] | 93.5 [74.2-113.1] | 90.9 [74.9-114.9] | 87.3 [71.7-108.6] |
| Previous in MI, n (%) | 168 (2.2%) | 67 (1.7%) | 21 (1.9%) | 17 (2.3%) | 13 (2.6%) | 50 (3.7%) |
| Obstructive stenosis (≥50%) | 1182(14.4%) | 0 (0%) | 146 (12.3%) | 144 (18.4%) | 189 (34.4%) | 703 (48.3%) |
| Calcium score 0 | 4145 (50.4%) | 3802 (89.5%) | 244 (20.5%) | 66 (8.4%) | 18 (3.3%) | 15 (1.0%) |
| Calcium score 1-9 | 750 (9.1%) | 177 (4.2%) | 362 (30.5%) | 137 (17.5%) | 51 ( 9.3%) | 23 (1.6%) |
| Calcium score 10-99 | 1580 (19.2%) | 147 (3.5%) | 483 (40.7%) | 409 (52.2%) | 253 (46.1%) | 288 (19.8%) |
| Calcium score 100-299 | 878 (10.7%) | 56 (1.3%) | 72 (6.1%) | 138 (17.6%) | 152 (27.7%) | 460 (31.6%) |
| Calcium score ≥300 | 872 (10.6%) | 66 (1.6%) | 27 (2.3%) | 34 (4.3%) | 75 (13.7%) | 670 (46.0%) |

SIS: Segment involvement score, CACS: Coronary artery calcium score, BMI: Body mass index, eGFR: Estimated glomerular filtration rate, MI: Myocardial infarction.

**Table S5b,** Patient characteristics in the SIS and CACS group

|  | **CACS group** | | | | | |
| --- | --- | --- | --- | --- | --- | --- |
| **Characteristics** | **Overall**, N = 8,225^1^ | **0**, N = 4,145^1^ | **1-9**, N = 750^1^ | **10-99**, N = 1,580^1^ | **100-299**, N = 878^1^ | **≥300**, N = 872^1^ |
| Age, median [25-75] | 58.0 [49.0-66.0] | 52.0 [44.0-60.0] | 59.0 [52.0-66.0] | 62.0 [55.0-68.0] | 65.0 [58.0-71.0] | 68.0 [61.0-73.0] |
| Male sex, n (%) | 4,074 (49.5%) | 1,730 (41.7%) | 409 (54.5%) | 818 (51.8%) | 507 (57.7%) | 610 (70.0%) |
| Smoking, current, n (%) | 850 (13.1%) | 403 (12.2%) | 74 (11.9%) | 155 (12.2%) | 118 (17.0%) | 100 (15.8%) |
| Diabetes, n (%) | 721 (9.5%) | 199 (5.2%) | 74 (10.5%) | 173 (11.9%) | 123 (15.2%) | 152 (19.5%) |
| Hypertension, n (%) | 3,152 (45.9%) | 1,177 (34.1%) | 333 (50.9%) | 717 (53.8%) | 434 (59.5%) | 491 (70.0%) |
| Hyperlipidemia, n (%) | 1,814 (27.5%) | 590 (17.6%) | 184 (29.4%) | 431 (34.0%) | 296 (42.1%) | 313 (48.4%) |
| BMI, median, [25-75] | 26.4 [23.7-29.7] | 26.0 [23.4-29.4] | 26.7 [23.9-29.9] | 26.6 [23.9-29.8] | 27.1 [24.3-30.4] | 26.8 [24.2-30.4] |
| eGFR, mL/min/1.73 m^2^ | 96.0 [77.0-118.5] | 101.6 [83.0-125.1] | 94.2 [75.8-118.8] | 90.2 [74.1-112.0] | 86.4 [71.1-109.2] | 85.7 [69.2-106.7] |
| Previous MI, n (%) | 168 (2.2%) | 62 (1.6%) | 18 (2.5%) | 23 (1.6%) | 30 (3.7%) | 35 (4.5%) |
| Obstructive stenosis (≥50%) | 1182 (14.4%) | 60 (1.4%) | 69 (9.2%) | 282 (17.8%) | 332 (37.8%) | 439 (50.3) |
| SIS 0 | 4248 (51.6%) | 3802 (91.7%) | 177 (23.9%) | 147 (9.3%) | 56 (6.4%) | 66 (7.6%) |
| SIS 1 | 1188 (14.4%) | 244 (5.9%) | 362 (48.3%) | 483 (30.6%) | 72 (8.2%) | 27 (3.1%) |
| SIS 2 | 784 (9.5%) | 66 (1.6%) | 137 (18.3%) | 409 (25.9%) | 138 (15.7%) | 34 (3.9%) |
| SIS 3 | 549 (6.7%) | 18 (0.4%) | 51 (6.8%) | 253 (16.0%) | 152 (17.3%) | 75 (8.6%) |
| SIS ≥4 | 1456 (17.7%) | 15 (0.4%) | 23 (3.1%) | 288 (18.8%) | 460 (52.4%) | 670 (76.8%) |

SIS: Segment involvement score, CACS: Coronary artery calcium score, BMI: Body mass index, eGFR: Estimated glomerular filtration rate, MI: Myocardial infarction.

**Table S6,** Risk of death or MI in relation to SIS and CACS when grouped equally sized groups

|  | **Unadjusted** | | **Adjusted*** | |
| --- | --- | --- | --- | --- |
|  | HR | 95%CI | HR | 95%CI |
| **SIS** | | | | |
| 0-1 | 1 | ref | 1 | Ref |
| 2 | 2.62 | 1.81-3.80 | 1.91 | 1.02-1.58 |
| 3 | 3.12 | 2.11-4.62 | 2.26 | 1.08-1.75 |
| ≥4 | 4.56 | 3.50-5.93 | 2.91 | 1.65-2.34 |
|  |  |  |  |  |
| **CACS** | | | | |
| 0-25 | 1 | Ref | 1 | Ref |
| 26-74 | 1.42 | 0.91-2.21 | 1.08 | 0.69-1.69 |
| 75-142 | 2.40 | 1.58-3.67 | 1.65 | 1.07-2.55 |
| ≥143 | 4.39 | 3.40-5.66 | 2.57 | 1.91-3.46 |

SIS: Segment involvement score, CACS: Coronary artery calcium score *Adjusted for age, sex, smoking, diabetes mellitus, hypertension, hyperlipidemia, body mass index, estimated glomerular filtration rate and previous myocardial infarction. Complete case analysis.

**Table S7,** Ability of segment involvement score (SIS), coronary artery calcium score (CACS) and combination of SIS and CACS (SIS x CACS) to discriminate between patients with subsequent composite of death or myocardial infarction, death alone and myocardial infarction alone.

|  | SIS | | CACS | | SIS x CACS | |
| --- | --- | --- | --- | --- | --- | --- |
|  | AUC | (95%CI) | AUC | (95%CI) | AUC | (95%CI) |
| Death or Myocardial infarction | 0.68 | (0.65-0.72) | 0.70 | (0.67-0.74) | 0.69 | (0.65-0.72) |
| Death | 0.67 | (0.63-0.70) | 0.70 | (0.66-0.74) | 0.68 | (0.64-0.72) |
| Myocardial infarction | 0.72 | (0.67-0.78) | 0.72 | (0.67-0.77) | 0.72 | (0.67-0.77) |
